# Draft whole-genome sequence of multidrug-resistant *Enterococcus faecium* strain MKL_BAU_Fe01 isolated from chicken meat

**DOI:** 10.1128/mra.00043-25

**Published:** 2025-04-30

**Authors:** Al Amin Howlader, FNU Nahiduzzaman, Raihan Islam Annan, Arnob Saha, Md. Ariful Islam, Mst. Minara Khatun

**Affiliations:** 1 Department of Microbiology and Hygiene, Bangladesh Agricultural University 54492, Mymensingh, Bangladesh; University of Maryland School of Medicine, Baltimore, Maryland, USA

**Keywords:** *Enterococcus*, food safety, nosocomial infection, chicken meat, *Enterococcus faecium*, multidrug resistance, public health, foodborne infection, urinary tract infection

## Abstract

We report the draft genome sequence of *Enterococcus faecium* MKL_BAU_Fe01 from chicken meat in Bangladesh. The 2,953,746 bp genome, with 37.99% GC content across 264 contigs, contains 3,117 genes, including 3,007 Coding Sequences (CDSs), 17 rRNAs, and 89 tRNAs, along with several antibiotic resistance and pathogenic genes.

## ANNOUNCEMENT

*Enterococcus faecium* is a commensal microorganism that can also act as an opportunistic pathogen due to its ability to acquire several virulence factors (1). This microorganism is commonly detected in various animal-derived food products, including meat, milk, and eggs, highlighting its widespread presence along the food production chain and its potential role in foodborne transmission (2, 3). Enterococci can cause infections like bacteremia, endocarditis, endophthalmitis, and urinary tract infections (4).

This study was conducted in nine different areas within a 5 km radius around 24.722880°N, 90.415595°E in the Mymensingh district, Bangladesh, from 1 August to October 2024. Chicken meat swab samples (*n* = 74) were collected from slaughtered broiler chickens at live bird markets using sterile cotton swabs. The samples were enriched overnight at 37°C in Nutrient Broth (HiMedia, India), subsequently streaked on Pfizer Selective *Enterococcus* Agar (PSEA; HiMedia, India) and incubated at 37°C for 24 h. *E. faecium* was detected in 12.16% of samples through culture, staining morphology, and PCR targeting the *ddl_E. faecium_* gene using the primer described by Dutka-Malen et al. (5). The PCR thermal profile included 94°C for 5 min, 30 cycles of 94°C for 30 s, 50°C for 90 s, and 72°C for 60 s, with a final extension at 72°C for 10 min.

The sequenced sample was collected at 24.7384°N, 90.4253°E on 16 September 2024 and selected for sequencing based on the phenotypic resistance against the highest number of antibiotics, including ciprofloxacin (5 µg), vancomycin (30 µg), tetracycline (30 µg), ampicillin (25 µg), erythromycin (15 µg), and cefotaxime (30 µg; HiMedia, India). Antimicrobial resistance patterns were identified using the disc diffusion technique (6).

It was transported to the laboratory located at 24.724469°N, 90.437250°E on 20 September 2024 for further analysis. Genomic DNA was extracted from the bacterial isolate on 21 September 2024, using the QIAcube Connect automated nucleic acid extractor (QIAGEN N.V., Germany). The isolate was obtained from a streaked bacterial culture plate prepared with PSEA, following incubation at 37°C for 24 h. DNA was sheared to ~350 bp using a Covaris ultrasonicator (Covaris, USA). Size selection was performed using AMPure XP beads (Beckman Coulter, USA) to retain DNA fragments within the desired size range. Sequencing libraries were prepared with the TruSeq DNA PCR-Free Library Prep Kit (Illumina, USA). Paired-end sequencing (2 × 150 bp) was conducted using the Illumina NextSeq-500 (Illumina, USA) to generate high-quality reads for bacterial whole-genome analysis.

Initially, 1,916,294 raw reads underwent quality checks and trimming using Trim Galore v.0.6.5dev (https://github.com/FelixKrueger/TrimGalore). Assembly was conducted using SPAdes v.4.0.0 (7), and assembly quality was evaluated with QUAST v.5.2.0 (8). Error correction with Pilon v.1.24 (9) yielded 264 contigs totaling 2,953,746 bp, with an average GC content of 37.99%, N50 of 106,686 bp, N90 of 13,902 bp, auN of 141,937.4 bp, and eight contigs in L50 and 36 in L90. For genome annotation, PGAP v.6.9 (10) was employed using NCBI database (Method: Best-placed reference protein set; GeneMarkS-2+). The annotation identified the following features in Table 1.

**TABLE 1:** Summary of the annotation features of *E. faecium* MKL_BAU_Fe01

Category	Count
Genes (total)	3,117
CDSs (total)	3,007
Genes (coding)	2,864
CDSs (with protein)	2,864
Genes (RNA)	110
rRNAs	17
Complete rRNAs	11
Partial rRNAs	6
tRNAs	89
ncRNAs	4
Pseudogenes (total)	143
CDSs (without protein)	143
Pseudogenes (ambiguous residues)	0
Pseudogenes (frameshifted)	50
Pseudogenes (incomplete)	60
Pseudogenes (internal stop)	54
Pseudogenes (multiple problems)	20

AMR and pathogenic genes were identified using AMRFinderPlus v.4.0.15 (11), CARD v.4.0.0 (12), and VFDB v.2.0 (13) revealing the presence of several resistance genes, including *eat(A)_T450I*, *copy*, *aac(6')-I*, and *msr(C)* (coverage: 95.97%–100%). Additionally, the virulence regulator *plcR* (85.61% coverage) was also detected.

## Data Availability

The whole-genome shotgun project for *Enterococcus faecium* MKL_BAU_Fe01 has been deposited at DDBJ/ENA/GenBank under the accession number JBKEKB000000000. The version described in this paper is JBKEKB010000000. It is available under BioProject PRJNA1201574 and BioSample SAMN45938320. Raw sequencing data are accessible in the SRA under accession SRR31822501.
